# P-553. Evaluation of the measles surveillance system in the Kyrgyz Republic, 2024

**DOI:** 10.1093/ofid/ofaf695.768

**Published:** 2026-01-11

**Authors:** Aigul Mamatova, Aisuluu Kubatova, Alfiya Denebayeva, Gulzhan Amanova, Roberta Horth, Dilyara Nabirova

**Affiliations:** Central Asia FETP, Bishkek, Bishkek, Kyrgyzstan; Ministry of Health of the Kyrgyz Republic, National Institute of Public Health, Bishkek, Kyrgyzstan, Bishkek, Bishkek, Kyrgyzstan; Central Asia FETP, Bishkek, Bishkek, Kyrgyzstan; Central Asia FETP, Bishkek, Bishkek, Kyrgyzstan; US Centers for Disease Control and Prevention, Dulles, Virginia; CDC Central Asia office, Almaty, Almaty, Kazakhstan

## Abstract

**Background:**

In 2023, the Kyrgyz Republic reported over 7,000 cases of measles, including nine fatalities. In 2022, an integrated electronic case reporting system for notifiable diseases, including measles, was introduced to enhance monitoring and response capabilities. This study aimed to evaluate the surveillance system, identify strengths and weaknesses, and develop actionable recommendations for improvement.

**Methods:**

Using the U.S. Centers for Disease Control and Prevention (CDC) updated surveillance system evaluation guidelines, we evaluated Kyrgyzstan’s measles surveillance system in February and April 2024. Two of ten healthcare centers and the Bishkek State Sanitary and Epidemiological Surveillance Center (SES) participated. Data were abstracted from the national electronic case reporting system, weekly paper-based case reporting forms, medical records, and reports from more than 1,700 medical organizations.

**Results:**

The electronic case reporting system recorded a total of 8,224 emergency notifications for measles, of which 7,046 were confirmed, including 2,308 cases in Bishkek. Case notification data were transmitted in real time from healthcare facilities, with 94% (2,170/2,308) of emergency notifications received by the SES within 12 hours of diagnosis. However, 7% (159/2,308) of these notifications contained errors in diagnosis dates. Of all emergency notifications, 92% (7,582/8,244) were investigated within 48 hours, and 2.1% (n=176) contained errors in registration dates. Of 4,245 laboratory samples collected, 78% had been taken within 4–28 days after the appearance of rash, 71% were received by the laboratory within 24 hours of collection, and 99% were tested within 24 hours.

**Conclusion:**

The evaluation indicates that the surveillance system effectively facilitates rapid notification of measles cases. However, weaknesses in data completeness, quality, and timeliness of sample collection were identified. Implementing additional data entry checks could enhance data quality, while further training for healthcare providers and laboratory personnel may help reduce delays in sample collection and transport, ultimately improving the overall effectiveness of the measles surveillance system in Kyrgyzstan.

**Disclosures:**

All Authors: No reported disclosures

